# Supplementary material for: Elemental Chemometrics as Tools to Depict Stalked Barnacle (Pollicipes pollicipes) Harvest Locations and Food Safety
Source: Molecules. 2022 Feb 15;27(4):1298. doi: 10.3390/molecules27041298 (PMC8874366; doi:10.3390/molecules27041298)
Supplement: Supplementary file 1 [file molecules-27-01298-s001.zip › molecules-1594646-supplementary.pdf]

Table S1. Average, maximum and minimum concentrations (mg/Kg ww) of all the analysed elements (N=15 per site).

	Sampling Site	Average ± Standard Deviation	Maximum	Minimum
As	VC	9.25 ± 3.26	18.12	5.58
	Mt	22.71 ± 10.96	42.63	7.38
	Er	36.54 ± 5.69	43.65	24.82
	CR	29.31 ± 13.82	53.51	0.00
	Si	30.55 ± 6.32	40.95	20.51
	Sa	33.48 ± 12.50	65.67	20.78
Br	VC	5.03 ± 1.74	8.90	2.64
	Mt	39.83 ± 34.54	103.99	3.72
	Er	82.61 ± 18.85	120.96	49.80
	CR	71.79 ± 20.22	129.03	44.68
	Si	87.27 ± 17.89	130.87	66.53
	Sa	69.78 ± 25.09	134.49	33.30
Ca	VC	275.18 ± 280.17	90.40	1257.60
	Mt	1329.11 ± 981.60	269.09	2912.77
	Er	2845.95 ± 873.35	1547.69	4744.91
	CR	2744.87 ± 1440.28	1108.06	6337.32
	Si	2011.18 ± 503.01	1024.83	3213.22
	Sa	1549.99 ± 551.41	741.38	3042.55
Cl	VC	174.07 ± 132.23	26.00	484.11
	Mt	1165.64 ± 1070.91	77.82	3102.90
	Er	3487.83 ± 865.10	1840.23	5466.28
	CR	2338.80 ± 793.28	1262.31	3695.97
	Si	3191.25 ± 639.92	2054.38	4579.49
	Sa	1880.68 ± 578.00	667.72	2617.69
Cr	VC	0.15 ± 0.51	2.01	0.00
	Mt	0.08 ± 0.16	0.55	0.00
	Er	0.35 ± 0.12	0.47	0.00
	CR	0.25 ± 0.22	0.69	0.00
	Si	0.33 ± 0.17	0.71	0.00
	Sa	0.29 ± 0.13	0.50	0.00
Cu	VC	0.40 ± 0.30	1.15	0.15
	Mt	0.64 ± 0.33	1.17	0.18
	Er	1.04 ± 0.22	1.55	0.71
	CR	1.18 ± 0.34	1.80	0.63
	Si	0.88 ± 0.21	1.22	0.59
	Sa	0.92 ± 0.16	1.30	0.62
Fe	VC	4.03 ± 2.37	10.44	2.37
	Mt	48.6 ± 55.7	197.3	2.45
	Er	83.5 ± 35.0	152.4	31.96
	CR	59.8 ± 19.5	103.3	28.12
	Si	30.6 ± 34.8	154.9	12.60
	Sa	72.2 ± 50.9	211.4	21.01
K	VC	7918.88 ± 2734.59	5539.83	14010.57
	Mt	8252.62 ± 3743.99	2162.72	18466.20
	Er	6063.08 ± 930.34	4115.47	7854.20
	CR	5787.91 ± 1220.96	4139.43	8071.57
	Si	4856.83 ± 1099.20	3181.55	7663.11

	Sa	4747.90 ± 1458.32	2733.60	6615.08
Mn	VC	0.20 ± 0.08	0.42	0.07
	Mt	1.43 ± 1.29	3.37	0.00
	Er	2.22 ± 0.82	4.05	1.33
	CR	2.28 ± 0.65	3.70	1.12
	Si	1.58 ± 0.66	2.95	0.82
	Sa	2.75 ± 2.42	9.28	0.74
Na	VC	3767.71 ± 888.84	4908.77	2314.39
	Mt	8306.10 ± 3447.80	13688.25	3762.97
	Er	13659.67 ± 1587.02	17230.17	10334.53
	CR	11938.62 ± 2267.30	17519.36	9295.05
	Si	12604.56 ± 1487.86	15398.32	10099.87
	Sa	11040.86 ± 1430.03	13638.63	8266.55
Ni	VC	0.09 ± 0.23	0.87	0.00
	Mt	0.19 ± 0.15	0.51	0.00
	Er	0.50 ± 0.15	0.73	0.26
	CR	0.36 ± 0.08	0.49	0.19
	Si	0.57 ± 0.10	0.82	0.43
	Sa	0.46 ± 0.21	0.89	0.19
P	VC	3116.78 ± 1679.18	6836.16	1774.34
	Mt	3319.26 ± 1182.82	5940.74	830.30
	Er	3210.93 ± 960.80	5288.25	1411.40
	CR	3715.79 ± 1434.52	7004.15	1613.73
	Si	2273.30 ± 669.56	3168.86	1009.68
	Sa	2404.05 ± 1319.31	5555.79	873.80
Pb	VC	0.56 ± 0.56	2.38	0.14
	Mt	0.57 ± 0.72	2.12	0.00
	Er	1.18 ± 0.76	2.73	0.00
	CR	1.62 ± 3.83	14.93	0.00
	Si	1.64 ± 2.44	7.73	0.00
	Sa	0.50 ± 0.61	1.54	0.00
Pr	VC	1.26 ± 2.45	9.03	0.00
	Mt	0.70 ± 1.81	6.50	0.00
	Er	0.04 ± 0.18	0.73	0.00
	CR	0.00 ± 0.00	0.00	0.00
	Si	0.00 ± 0.00	0.00	0.00
	Sa	0.12 ± 0.36	1.33	0.00
Rb	VC	3.27 ± 0.83	4.87	2.20
	Mt	5.27 ± 2.75	11.49	2.89
	Er	8.37 ± 2.65	12.13	2.65
	CR	6.69 ± 3.78	14.87	3.43
	Si	5.17 ± 3.91	10.33	0.00
	Sa	5.98 ± 3.41	12.37	2.64
Ru	VC	7.37 ± 23.58	90.56	0.00
	Mt	6.95 ± 19.87	72.31	0.00
	Er	25.08 ± 48.08	140.29	0.00
	CR	5.96 ± 19.06	73.19	0.00
	Si	10.86 ± 32.47	121.86	0.00
	Sa	16.85 ± 24.78	66.46	0.00
S	VC	2673.77 ± 1349.72	5509.52	1462.34
	Mt	5299.83 ± 2668.68	8978.58	1705.88

	Er	9385.13 ± 2440.91	13929.72	4295.80
	CR	7859.81 ± 2905.67	12567.87	3440.57
	Si	7282.24 ± 1846.21	9957.40	3436.99
	Sa	6648.98 ± 2594.75	10451.11	2166.75
Se	VC	0.61 ± 0.13	0.92	0.40
	Mt	0.95 ± 0.22	1.40	0.51
	Er	0.87 ± 0.13	1.11	0.63
	CR	0.71 ± 0.18	1.06	0.49
	Si	0.49 ± 0.08	0.64	0.32
	Sa	0.64 ± 0.13	0.91	0.39
Sm	VC	0.73 ± 1.49	5.65	0.00
	Mt	0.62 ± 0.96	2.47	0.00
	Er	0.00 ± 0.00	0.00	0.00
	CR	0.00 ± 0.00	0.00	0.00
	Si	0.00 ± 0.00	0.00	0.00
	Sa	0.00 ± 0.00	0.00	0.00
Sr	VC	1.34 ± 1.83	7.59	0.22
	Mt	25.75 ± 24.57	65.09	1.76
	Er	53.72 ± 14.19	82.11	28.45
	CR	51.98 ± 34.66	157.77	21.84
	Si	49.16 ± 9.44	66.66	34.18
	Sa	39.63 ± 8.78	59.76	28.85
Ti	VC	0.35 ± 0.45	1.29	0.00
	Mt	1.10 ± 1.74	6.65	0.00
	Er	1.11 ± 1.67	6.40	0.00
	CR	1.09 ± 1.28	4.89	0.00
	Si	0.28 ± 0.58	1.87	0.00
	Sa	0.72 ± 0.77	2.71	0.00
V	VC	0.00 ± 0.00	0.00	0.00
	Mt	0.00 ± 0.00	0.00	0.00
	Er	0.00 ± 0.00	0.00	0.00
	CR	0.46 ± 0.56	1.91	0.00
	Si	0.27 ± 0.39	1.20	0.00
	Sa	0.03 ± 0.13	0.51	0.00
Y	VC	3.24 ± 1.58	5.57	0.00
	Mt	4.10 ± 3.13	10.62	0.00
	Er	1.51 ± 3.18	8.73	0.00
	CR	3.37 ± 3.85	8.97	0.00
	Si	1.07 ± 2.84	8.77	0.00
	Sa	3.93 ± 3.41	8.38	0.00
Zn	VC	5.16 ± 1.75	10.60	3.46
	Mt	89.5 ± 82.8	189.7	3.93
	Er	304.8 ± 88.6	571.0	215.1
	CR	237.0 ± 55.5	356.5	173.9
	Si	231.5 ± 50.6	377.0	142.5
	Sa	191.9 ± 53.5	332.3	121.8
